# Lipid Phases and Cell Geometry During the Cell Cycle of *Streptococcus pneumoniae*

**DOI:** 10.3389/fmicb.2019.00351

**Published:** 2019-03-18

**Authors:** Philippe Calvez, Juliette Jouhet, Véronique Vié, Claire Durmort, André Zapun

**Affiliations:** ^1^Univ. Grenoble Alpes, CNRS, CEA, IBS, Grenoble, France; ^2^UMR 5168 CNRS, CEA, INRA, CEA Grenoble, Laboratoire de Physiologie Cellulaire Végétale, Bioscience and Biotechnologies Institute of Grenoble, Université Grenoble Alpes, Grenoble, France; ^3^Univ Rennes, CNRS, IPR-UMR 6251, ScanMat-UMS2001, Rennes, France

**Keywords:** bacterial membrane, lamellar phases, bacterial division, lipid domains, bacterial morphogenesis

## Abstract

The coexistence of different lipid phases is well-known *in vitro*, but evidence for their presence and function in cellular membranes remains scarce. Using a combination of fluorescent lipid probes, we observe segregation of domains that suggests the coexistence of liquid and gel phases in the membrane of *Streptococcus pneumoniae*, where they are localized to minimize bending stress in the ellipsoid geometry defined by the cell wall. Gel phase lipids with high bending rigidity would be spontaneously organized at the equator where curvature is minimal, thus marking the future division site, while liquid phase membrane maps onto the oblong hemispheres. In addition, the membrane-bound cell wall precursor with its particular dynamic acyl chain localizes at the division site where the membrane is highly curved. We propose a complete “chicken-and-egg” model where cell geometry determines the localization of lipid phases that positions the cell division machinery, which in turn alters the localization of lamellar phases by assembling the cell wall with a specific geometry.

## Introduction

Membrane curvatures can induce partitioning of lipid phases. *In vitro*, liquid disordered phase has been observed in higher-curvature regions compared to ordered phase which preferentially segregates into more planar regions, suggesting a possible interplay between cell geometry and lipid domain organization (Baumgart et al., 2003). However, evidence for coexisting lamellar phases in cells remain scarce and their roles largely unexplored, particularly in bacteria (Bagatolli and Mouritsen, 2013; Barák and Muchová, 2013). Bacterial cell division, where the membrane supports the assembly of the cell-wall while being subject to large geometrical constrains induced by the cell-wall itself, is a perfect system to investigate possible functions of the lipid bilayer.

The main conserved element of the division process is the polymerization of FtsZ as a cytoplasmic circumferential Z-ring, where it scaffolds other division proteins (Jacq et al., 2015; Haeusser and Margolin, 2016). Correct positioning of the Z-ring depends on various factors (Monahan et al., 2014; Garcia et al., 2016; Haeusser and Margolin, 2016; Kretschmer and Schwille, 2016), but the underlying physicochemical cues are not known. In rod-shaped bacteria, it has been proposed that the heterogeneous distribution of specific phospholipids and regions enriched in anionic head groups may contribute to the choice of the division site (Mileykovskaya and Dowhan, 2005), although the mechanisms driving membrane heterogeneity are unknown. Fluid polyunsaturated acyl chains have been observed at the division site in *Shewanella livingstonensis* (Sato et al., 2012), but this intriguing observation was not considered with respect to the cell cycle.

As a Gram-positive organism *Streptococcus pneumoniae* is a good model to study membrane during division as its cell cycle is well-characterized regarding cell-wall assembly (Zapun et al., 2008; Massidda et al., 2013). The membrane composition is simple with a majority of glucosyldiacylglycerol (GDG) and galactosylglucosyldiacylglycerol (GGDG), while phospholipids consist mostly of cardiolipin (CL) and phosphatidylglycerol (PG) (Brundish et al., 1967; Trombe et al., 1979). Glycolipids and phospholipids are organized in gel (L_β_) phase below their melting temperature θ_m_, in liquid crystalline (L_α_) phase above this transition temperature. Within one lipid class, the θ_m_ depends mainly on the length and the degree of unsaturation of the acyl chains. Greater length favors van der Waals interactions between acyl chains responsible for the rigidity of L_β_-phase while unsaturation favors the fluidity of L_α_-phase by introducing kinks in the acyl chain (Oldfield and Chapman, 1972). *S. pneumoniae* lacks the genes necessary for the biosynthesis of hopanoids (Malott et al., 2012), so that only L_α_ and L_β_-phases might coexist in its membrane. Finally, neither sphingolipids nor flotillin-like proteins, which are known to influence lipid domains organization (Bach and Bramkamp, 2013; Sohlenkamp and Geiger, 2016) are present in this bacterium. Thus, phase sorting could potentially result solely from curvature stresses generated by the cell-wall and the Z-ring constriction. Conversely, self-lateral organization of the membrane could underlie the localization of the division site. In this study, a combination of lipid dyes was used to investigate the organization of the plasma membrane during the cell cycle of *S. pneumoniae*.

## Materials and Methods

### Fluorescent Probes and Lipids

Nonyl acridine orange [3,6-*bis*(Dimethylamino)-10-nonyl-acridinium bromide] (NAO) and FM4-64 were purchase from Molecular Probes. 1,2-Dioleoyl-*sn*-glycero-3-phosphoethano lamine-*N*-(lissamine rhodamine B sulfonyl) (DOPE-rho), 1,2-myristoyl-*sn*-glycero-3-phosphoethanolamine-*N*-(7-nitro-2-1,3-benzoxadiazol-4-yl) (DMPE-NBD); 1-palmitoyl-2-{12-[(7-nitro-2-1,3-benzoxadiazol-4-yl)amino]dodecanoyl}-*sn*-glycero-3-phos pho-glycerol (NBD-PG), 1,2-dioleoyl-sn-glycero-3-phospho-(1′-rac-glycerol) (DOPG), 1,2-dipalmitoyl-sn-glycero-3-phospho-(1′-rac-glycerol) (DPPG) and 1-palmitoyl-2-oleoyl-*sn*-glycero-3-phospho-(1′-rac-glycerol) (POPG) were purchased from Avanti polar lipids. NBD-labeled lipid II was a generous gift from E. Breukink (van Dam et al., 2007).

### Strains and Growth Conditions

*Streptococcus pneumoniae* strains R800 (R6 *rpsL1*; Str^R^) (Sung et al., 2001) and derivatives expressing green (R800 *ftsZ*::*ftsZ*-*GFP*; Str^R^) (Fleurie et al., 2014) or red fluorescent FtsZ (R800 *ftsZ*::*ftsZ*-*mKate*; Str^R^) (Bonnet et al., 2017) were grown at 30°C with 5% CO_2_ in C-medium supplemented with 0.5% yeast extract (CY) (Lacks and Hotchkiss, 1960). For lipid analysis, strain R800 was grown in Todd Hewitt medium supplemented with 0.5% yeast extract.

### Microscopy

Bacteria were imaged using an automated inverted epifluorescence microscope Nikon Ti-E/B equipped with the “perfect focus system” (PFS, Nikon), a phase contrast objective (CFI Plan Apo Lambda DM 100X, NA1.45), Semrock filter sets for GFP (Ex: 482BP35; DM: 506; Em: 536BP40) and mCherry (Ex: 562BP40; DM: 593; Em: 640BP75), a LED light source (Spectra X Light Engine, Lumencor) and a cCMOS camera (Neo sCMOS, Andor). Image acquisition was performed using the Volocity software package. Images were analyzed using the open-source software Oufti (Paintdakhi et al., 2016). Cells were automatically detected using the phase contrast image and delineated to allow visual inspection, which permitted disambiguation when cells were juxtaposed or forming chains. The long axis of each cells was determined automatically for subsequent calculation. The individual cell lengths were automatically determined using the objective and camera characteristics. To construct demographs, the fluorescence signal was normalized for each individual cell between the maximal and minimal value for this particular cell.

### Labeling

For NAO labeling, 950 μL of cell suspension were added to 50 μL of dye (200 mM in DMSO), for FM4-64 labeling, 1 mL of cell suspension was added to 10 μL of dye (0.2 mg mL^-1^ in DMSO). After incubation for 5 min at 4°C and three washes in CY, cells were resuspended in CY and observed immediately.

Fluorescent lipids were added in a ratio of 1:300 to the cellular lipids of the cell suspension, assuming the lipid concentration to be 8 μM in a culture of *S. pneumoniae* at an optical density of 1 at 600 nm. This estimate was based on a membrane surface area (two sides) of 7.3 μm^2^ half-occupied by lipids, a surface area per lipid molecule of 0.76 nm^2^ and a cell density of 10^9^ cells mL^-1^. Lipid probes in chloroform were dried under nitrogen in glass vials and resuspended in ethanol at the desired concentration (0.8 or 8 μM, 10^2^-fold final). Cultures (1 mL) in exponential growth phase at an optical density of 0.3 at 600 nm were then added to 10 μL of the lipid probe ethanol solution at room temperature. With DMPE-NBD, the probe/lipid ratio was 10:300 and the probe ethanol solution was prewarmed at 51°C. Resuspension was performed with three pipetting aspirations, followed by vortexing for 3 s. Cells were washed three times with CY and resuspended in CY. Cells were immediately observed or incubated further for chase experiments. In experiments with fixed cells and fluorescent lipid II, cells were fixed 40 min at room temperature with 1% paraformaldehyde prior to washing and labeling.

A limitation of the labeling methods used in this study is the fact that only the outer leaflet of the membrane bilayer is expected to be labeled. Unless flippases rapidly homogenize the two sides of the membrane, lipid probes added in the medium are expected to insert in the outer leaflet. The data were interpreted with the assumption that the composition of both the inner and outer leaflets were identical or similar.

Using lipid probes, the fraction of labeled cells and the background noise were highly susceptible to the handling of reagents and cells, and the exact procedure that was followed. Indeed, as the probes are not soluble in water and can spontaneously self-organize, the successful insertion in the cellular membrane depends on the concentrations, temperatures and mixing time, to the extent that pipetting speed certainly influenced labeling homogeneity. We also observed that excess pressure applied on the microscopy coverslip would alter the labeling patterns.

### Spheroplast Preparation

Spheroplasts were prepared by resuspending the pellet from 1 mL of cells at an OD_600_ of 0.3 in 50 μL of solution containing 200 mM MES (pH 6.5), 0.5 M sucrose, 20 mM MgCl_2_, 0.5 mg mL^-1^ lysozyme and 0.5 mg mL^-1^ LytA. Cells were incubated 60 min at 37°C followed by overnight storage at 4°C. Spheroplasts were also prepared from fixed cells in the same manner.

### Lipid Analysis

The cell cultures were diluted twofold when the optical density at 600 nm reached 0.15. The dilution was repeated three times to reach a steady state. Cultures were then left to grow and when they reached optical densities at 600 nm of 0.3, 0.7 and 1, about 10^11^ cells were pelleted at 25°C and freeze-dried in glass tubes before resuspension in 5 mL CHCl_3_. After addition of 2 mL of 150 mM NaCl and thorough mixing, phases were separated by centrifugation 10 min at 2000 *g* and the organic phase was extracted using a glass pipette. The organic extraction was repeated once with 5 mL CHCl_3_. Chloroform was then evaporated under N_2_ or Ar. Lipids were solubilized in 300 μL of CHCl_3_ and stored at -20°C.

The acyl chain composition was determined by gas-chromatography. Twenty mL of lipid extract were mixed with 10 μg of standard (C15:0, Sigma-Aldrich) and 3 mL of H_2_SO_4_ 2.5% in methanol were added, thoroughly mixed and incubated 1 h at 100°C. The reaction was stopped by the addition of 3 mL H_2_O. Fatty acid methyl esters were extracted with twice 3 mL of hexane. The hexane phase was analyzed by a GC-FID (Perkin-Elmer) on a BPX70 (SGE) column. Fatty acid methyl esters were identified by comparison of their retention times with those of the standard C15:0 and quantified by the surface peak method using 15:0 for calibration. Extraction and quantification were performed three times.

Lipids were isolated according to their polar head group using two-dimensional thin layer chromatography. Hundred μL of lipid extract were deposited onto 20 cm × 20 cm thin layer of silica (silica gel 60, Merck). The first solvent was chloroform:methanol:water (65:25:4, v/v) and the second was chloroform:acetone:methanol:acetic acid:water (50:20:10:10:5, v/v). After spraying with 2% (v/v) 8-anilino-1-naphthalenesulfonic acid in methanol, lipids were then visualized under UV light and scraped off the plate. Lipids were identified by mass spectrometry and quantified by gas-chromatography as described previously (Abida et al., 2015).

For ion trap MS analyses, purified lipid classes were dissolved in 10 mM ammonium acetate in pure methanol. They were introduced by direct infusion (ESI-MS) into a trap type mass spectrometer (AmazonXL, Bruker) and identified by comparison with standards. In these conditions, the produced ions were mainly present as H^-^, H^+^, NH_4_^+^, or Na^+^ adducts. Lipids were identified by MS analysis with their precursor ion or by neutral loss analyses after low energy collision-induced dissociation, as previously described (Gros and Jouhet, 2018).

### Surface Pressure Isotherms

Isotherms were recorded in triplicates on a 600 cm^2^ Langmuir-Blodgett Teflon trough (type 611, NIMA Technology) with a movable barrier using a Wilhelmy plate and a NIMA detector. Thirty-five μg of lipid extract from cells in exponential growth or late stationary phase were spread at the surface of nanopure water. The lipid film was equilibrated until evaporation of the organic solvent and compressed with a barrier velocity of 50 cm^2^ min^-1^. The bath temperature was measured using a thermocouple thermometer.

### AFM of Mixed Monolayers

Phospholipid monolayers were prepared on a 716 cm^2^ Langmuir-Blodgett Teflon trough (type 601BAM, NIMA Technology). Phospholipids were spread on nanopure water at the liquid/air interface between movable barriers using a high precision Hamilton micro-syringe. After 10 min to allow evaporation of the solvent, films were compressed by the moving barriers at a rate of 20 cm^2^ min^-1^ up to 20 mN m^-1^. Surface pressure was measured using a Wilhelmy plate and a NIMA detector. The peptide solution (1 mg mL^-1^, in 50 mM HEPES, pH 7.5, 100 mM NaCl) was injected in the sub-phase to reach a final concentration of 5 μg mL^-1^. The 95% pure peptide (H_2_N-ASQNKPKLADRFRGLIGSMFDE-OH) was from PeptLab (Université de Cergy-Pontoise, France). Once surface pressure equilibrium was reached, the film was transferred to a freshly cleaved mica support previously immersed in the sub-phase following the Langmuir–Blodgett method. The transfer was realized at a constant surface pressure and the speed was 0.16 mm min^-1^. The transfer surface pressure was 20.0 ± 0.5 mN m^-1^ for the DOPG/DPPG monolayer and 24.6 ± 0.5 mN m^-1^ in the presence of the peptide. Height maps were acquired in air at a scan speed of 1 Hz over 25 μm × 25 μm in ScanAsyst mode on a Nanoscope V MultiMode 8 with a silicon nitride tip (Bruker) with a stiffness constant of 0.06 N m^-1^. Images are representative of several places on the mica plates and the experiments were repeated twice.

### Red Edge Excitation Shift Measurements

NBD-PG in ethanol and DPPG or POPG in CHCl_3_ were mixed in a glass vessel and dried under N_2_ stream. After further drying overnight under vacuum, lipids were resuspended in 1.5 mL of 50 mM HEPES, pH 7.5, 150 mM NaCl, with or without 10 mM MgCl_2_, to reach final concentration of 1 mM lipids and 10 μM NBD-PG. Samples were freeze-thawed thrice and large uni-lamellar vesicles were prepared by extrusion through filters with 200 nm pores using an Avanti Polar Lipids Mini-Extruder. With DPPG, the whole procedure was carried out at 50°C. Samples were stored overnight in the dark at room temperature prior to recording emission spectra at temperature ranging from 20 to 50°C while varying the excitation wavelength from 435 to 505 nm in a Jasco FP-8500 fluorescence spectrometer.

## Results

**Figure 1:**
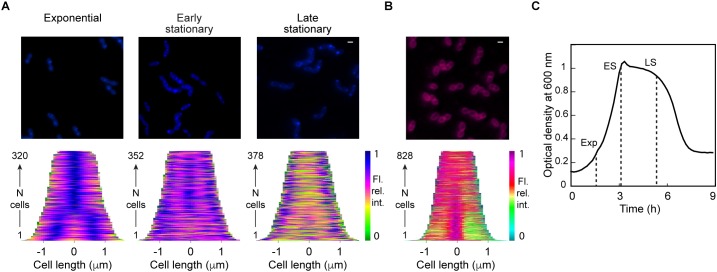
*Streptococcus pneumoniae* membrane characterization. **(A)** Cells at different stages of culture stained with NAO (blue) and the corresponding demographs showing the distribution of the fluorescent signals along the main cell axis in the population. **(B)** Cells stained with FM4-64 (magenta) and the corresponding demograph. The scale bar is 1 μm. Note that colors do not reflect the emission wavelength of the fluorophores but were chosen to ensure consistency across the different experiments and figures. **(C)** Typical growth curve of *S. pneumoniae* in CY-medium at 37°C. The dashed lines indicate the analyses during the exponential growth (Exp), and the early and late stationary phases (ES and LS, respectively).

**Figure 2:**
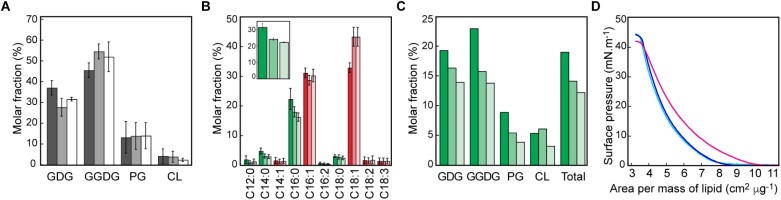
*Streptococcus pneumoniae* membrane composition. Lipid analysis was performed during the exponential growth phase (deep coloring), early and late stationary phase (middle and light coloring, respectively). **(A)** Relative amount of lipids by polar head groups; GDG, glucosyldiacylglycerol; GGDG, galactosylglucosyldiacylglycerol; PG, phosphatidylglycerol; and CL, cardiolipin. **(B)** Relative amount of acyl chains, saturated in green, unsatured in red. The inset show the total relative amount of saturated acyl chain. Error bars are the standard deviation of three independent experiments. **(C)** Relative amount of fully saturated lipids. **(D)** Surface pressure isotherms of monolayers prepared with lipid extract from exponentially growing cells recorded at 30°C (cyan), 37°C, (blue), and 43°C (magenta).

The dye 10-*N*-nonyl acridine orange (NAO), which binds the phosphate group of anionic phospholipids (Oliver et al., 2014), has revealed negatively charged membrane regions at the poles and division sites of bacilli (Mileykovskaya and Dowhan, 2000; Kawai et al., 2004). As a first step to characterize the plasma membrane of *S. pneumoniae*, we labeled cells with NAO or the non-specific membrane dye FM4-64 in exponentially growing cells and during the early and late stationary phase (Figure 1A–C). Micrographs were subjected to demograph analysis. Demographs are constructed by first integrating the fluorescence signal in each longitudinal segment of each individual cell and then normalizing the fluorescence in each cell by the maximum integrated value of a particular segment. Cells are then sorted by their length and the fluorescence values are plotted as a heat map (Paintdakhi et al., 2016). In exponentially growing *S. pneumoniae*, NAO staining was different from the signal of FM4-64 and stronger at the division site, showing that the division site is enriched in anionic lipids. During stationary phase, NAO showed gradual accumulation at the poles. These data indicate that cell division in *S. pneumoniae* is associated with a specific membrane organization, although no change in the proportion of the different head groups was observed between the exponential growth and the stationary phase (Figure 2A).

The acyl chain composition of polar lipids was also assessed (Figure 2B). Unexpectedly, the proportion of saturated chains decreased from 32% in the exponential growth phase to 24–22% in the subsequent non-growth phases. The larger amount of saturated acyl chains during exponential growth challenges the idea that cell division is directly related to membrane fluidity. Since the existence of a gel phase domain would be favored by high proportion of fully saturated lipids, we analyzed by mass spectrometry the distribution of unsaturations in the different lipid classes of *S. pneumoniae*. Complete results are given in Supplementary Figure S1 and Supplementary Tables S1–S4. The proportion of fully saturated lipids was found to account for nearly a fifth of the membrane during the exponential growth phase, decreasing to 12% in the late stationary phase, as shown in Figure 2C. Such a high proportion of saturated lipids is compatible with the segregation of lipid phases in the membrane of the pneumococcus. Surface pressure isotherms of monolayers prepared with lipid extracts from exponentially growing *S. pneumoniae* also suggest the presence of some L_β_-phase in dividing cells (Figure 2D). Isotherm spans were very similar at 30 and 37°C (5.14 ± 0.08 and 4.28 ± 0.31 cm^2^ μg^-1^, respectively) whereas they are shifted to larger molecular areas at 43°C (6.49 ± 0.29 cm^2^ μg^-1^, error is standard deviation of three measurements). This expansion of the lipid monolayer with increased temperature, could be the result of some domains undergoing an L_β_ to L_α_-phase transition between 37 and 43°C. This is consistent with the large proportion of palmitoyl chain (16:0) in *S. pneumoniae* (Figure 2B) and the known θ_m_ = 41°C of 1,2-dipalmitoyl-*sn*-glycero-3-phosphoglycerol (DPPG) (Zhang et al., 1997). Surface pressure isotherms of monolayers prepared with lipid extracts from cells in late stationary phase were similar below (25°C) and above (43°C) the DPPG phase transition temperature, with a change in molecular areas comparable to that observed at 43°C with lipids from exponentially growing cells (Supplementary Figure S2). This observation is consistent with the lower amount of saturated acyl chains in non-growing cells. No shelf was observed in the isotherm curves, as is typical from the expanded to condensed phase transition with pure lipids or ideal mixtures. However, with some mixtures such as 1-palmitoyl-2-oleoyl-sn-glycero-3-phosphoglycerol (POPG)-DPPG, the coexistence of phases is not visible as an inflection of the isotherms (Takamoto et al., 2001). In natural extracts, the complexity of the composition may obscure the phase transition signal, in particular in the presence of a diverse and abundant expanded liquid phase.

**Figure 3:**
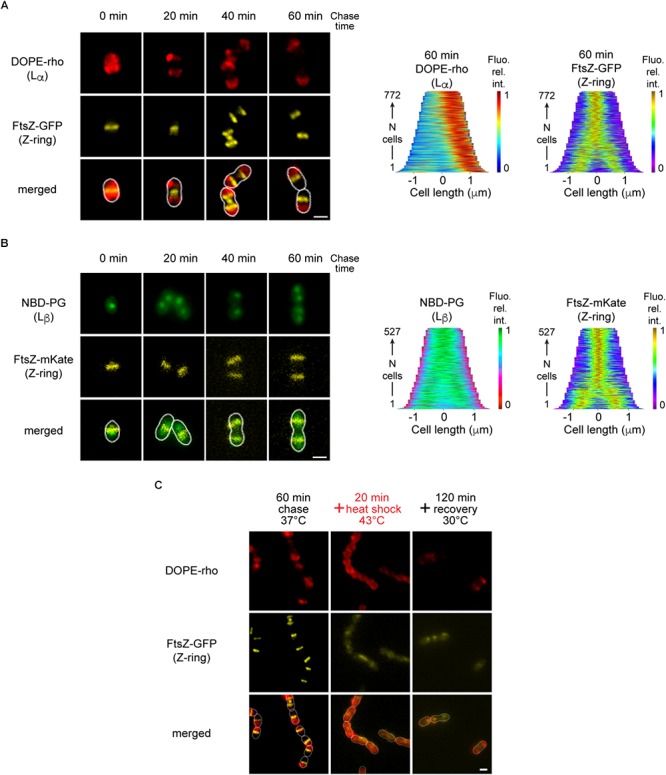
Localization of lipid-phases in *S. pneumoniae*. **(A)** Growing cells expressing FtsZ-GFP (yellow) labeled with fluorescent DOPE-rho to reveal L_α_-phase (red) imaged after various chase time and the demograph showing the distribution of the fluorescent signals along the main cell axis in the population after 60 min of chase. **(B)** Growing cells expressing FtsZ-mKate (yellow) labeled with fluorescent NBD-PG to reveal L_β_-phase domains (green) and the corresponding demographs. **(C)** DOPE-rho-labeled pulse-chased cells (red) expressing FtsZ-GFP (yellow) were submitted to a heat-shock prior to recovery. The scale bar is 1 μm. In merged images, the cell contour determined from phase contrast images is shown as a white line. Note that colors do not reflect the emission wavelength of the fluorophores but were chosen to ensure consistency across the different experiments and figures.

We then investigated if fluorescent phospholipids could reveal the segregation of lamellar phases. The unsaturated (18:1) 1,2-dioleoyl-*sn*-glycero-3-phosphoethanolamine-*N*-(lissamine rhodamine B sulfonyl) (DOPE-rho) was used to observe the distribution of L_α_-phases in cells expressing a green-fluorescent FtsZ. Cells were imaged immediately after a labeling pulse and during a chase; demographs show the distribution of fluorescence along the longitudinal axis of the cells in the population at different times (Figure 3A and Supplementary Figure S3A). The initial uniform DOPE-rho labeling remained localized on the parental hemispheres that parted during the chase, implying that a barrier at the equator prevents the lateral diffusion of the probe and the underlying L_α_-phase.

Conversely, the saturated (14:0) fluorescent lipid 1,2-dimyri stoyl-*sn*-glycero-3-phosphoethanolamine-*N*-(7-nitro-2-1,3-benz oxadiazol-4-yl) (DMPE-NBD) showed an equatorial localization when used to label a strain expressing a red fluorescent FtsZ (Supplementary Figure S4A), suggesting the presence of an L_β_-phase between the two hemispheres. Consistent labeling across microscopy slides was not obtained with DMPE-NBD. When inserting lipid probes into biological membranes from the aqueous medium, there is kinetic competition between the desired insertion and self-association of the probe. Balancing the desired and undesired outcome by adjusting the concentrations, temperature, and mixing procedure is difficult and could not be achieved reproducibly with DMPE-NBD.

The alternative saturated asymmetric (16:0/12:0) lipid 1-pal mitoyl-2-{12- [(7-nitro-2-1,3-benzoxadiazol-4-yl)amino]dode canoyl}-*sn*-glycero-3-phospho-glycerol (NBD-PG) used to label a strain expressing red fluorescent FtsZ (Figure 3B) displayed equatorial localization. Indeed, demographs show that labeling remained at a constant distance from the parental pole, confirming the equatorial localization of this dye, which likely signals the presence of L_β_-phase. Despite the equatorial localization revealed by the demographs, the DMPE-NBD and NBD-PG probes did not produce the typical dumbbell-shaped labeling expected for an equatorial ring (see the FtsZ-ring in Figure 3A for example). We propose that the probes cannot insert within the gel phase belt, but instead form independent domains that stack against the equatorial belt (Supplementary Figure S4B).

The NBD-PG probe has not been thoroughly studied, but some data are available regarding the closely related (16:0:12:0) NBD-PC (phosphatidylcholine). This probe showed uniform labeling of giant unilamellar vesicles of 1,2-dipalmitoyl-*sn*-glycero-3-phosphocholine (DPPC) and 1,2-dioleoyl-*sn*-glycero-3-phosphocholine (DOPC), with an elevated fluorescence λ_max_ in the later. In DPPC liposomes (16:0:12:0) NBD-PC was found to exhibit a significant red edge excitation shift of 4 nm above the phase transition temperature of DPPC. These observations were interpreted as indication that the fluorophore at the extremity of the dodecanoyl chain is deep inside the gel phase bilayer, whereas the acyl chain is likely folded back to expose the fluorophore at the lipid-water interface. To characterize the NBD-PG probe, we performed similar red edge excitation shift measurements in DPPG or POPG large unilamellar vesicles. A 4 nm shift of λ_max_ was observed at all temperature when increasing the excitation wavelength from 435 to 505 nm, indicating that the fluorophore lies at the lipid-water interface, even in DPPG membranes (Supplementary Figure S5). Therefore, NBD-PG in anionic lipid membranes behaves differently than NBD-PC in neutral PC membranes. Whether NBD-PG labels L_β_-phases remains an open question, but the different patterns observed with NBD-PG and DOPE-rho, as well as the identical labeling obtained with NBD-PG and DMPE-NBD, indicate that NBD-PG is at least segregated from the hemispheric L_α_-phases.

We have tested the impact of the possible phase transition detected *in vitro* by the surface pressure isotherms between 37 and 43°C (Figure 2D) on the segregation of the L_α_-phases *in vivo*. Cells expressing a green fluorescent FtsZ were pulse-labeled with DOPE-rho to reveal the L_α_-phase and chased over a generation time at 37°C to allow segregation of the labeled hemispheres. After shifting the temperature to 43°C for 20 min, the hemispheric DOPE-rho labeling spread over the whole bacterial surface (Figure 3C and Supplementary Figure S3B), as expected if the melting of an L_β_-phase barrier would allow lipid mixing over the whole cell surface. When cells were returned at 30°C and left to grow further for 1 h, a new segregation of the DOPE-rho labeled hemispheres was initiated. This effect is compatible with the organization of L_β_-phases at the equators in belts that are responsible for hemispheric segregation of L_α_-phases, although other cellular mechanisms, including changes of the lipid compositions cannot be ruled out on the time scale of the experiment.

**Figure 4:**
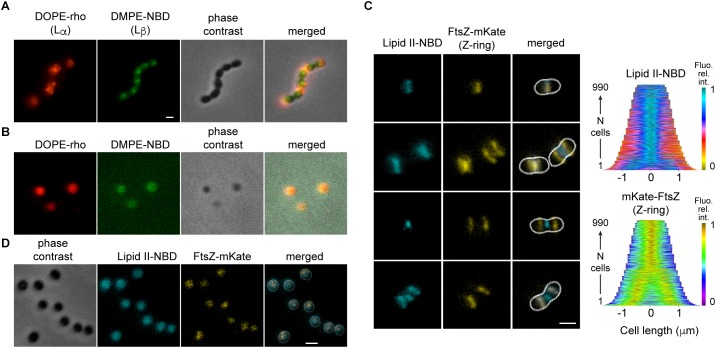
Localization of lipids in *S. pneumoniae* depends on the cell shape. **(A)** Cells were labeled simultaneously with DOPE-rho (red) and DMPE-NBD (green) prior to further growth for 60 min. **(B)** The cell wall of cells labeled and chased as in **(A)** was digested by peptidoglycan hydrolases. **(C)** Growing cells expressing FtsZ-mKate (yellow) labeled with fluorescent lipid II-NBD (cyan) and the corresponding demographs. **(D)** The cell wall of fixed cells labeled with lipid II-NBD was digested by peptidoglycan hydrolases.

Simultaneous insertion of DMPE-NBD and DOPE-rho confirmed the segregation of two distinct lipid phases in the membrane of dividing *S. pneumoniae* with an equatorial L_β_-phase preventing the diffusion of lipids between the hemispheric L_α_-phases (Figure 4A). When the cell-wall of such labeled cells was removed by enzymatic digestion, the segregation of lipid phases was abolished in the resulting spheroplasts (Figure 4B), indicating that the cell-wall-induced membrane geometry may govern the distribution of lipid phases as reported *in vitro* in pearling state liposomes for ordered and disordered phases of lipid (Baumgart et al., 2003). Indeed, streptococci are prolate ellipsoids with the smallest curvature region defining the equator that should accommodate the high bending rigidity of L_β_-phase whereas the low bending rigidity of L_α_-phase is suited for the larger curvature constraint of the oblong hemispheres.

The cell-wall precursor lipid II is a minor component of the membrane (<1 mol%) with peculiar properties (Kramer et al., 2004). Its large disaccharide pentapeptide head-group, which constitutes the cell-wall unit, is linked via a pyrophosphate to a long prenoyl undecamer with very fluid and dynamic properties (Ganchev et al., 2006; Chugunov et al., 2013). When added to growing cells (Figure 4C), NBD-labeled lipid II localized spontaneously to active division sites undergoing constriction. Fluorescent lipid II was not observed at equators where FtsZ is already observed prior to constriction. This localization pattern is similar to the localization of active cell-wall cross-linking, or that observed with fluorescent vancomycin that binds to lipid II or nascent cell-wall (Tsui et al., 2014). The same pattern of fluorescence was obtained when lipid II-NBD was added to fixed cells suggesting that its localization is not driven by the cell-wall building enzymatic activities but by the membrane physical properties or geometry (Supplementary Figure S6). Fluorescent lipid II labeled uniformly spherical cells prepared by digestion of the cell wall (Figure 4D). Flexibility of the undecaprenyl chain probably drives the accumulation of lipid II at the sites of greatest curvature stresses generated by constriction of the Z-ring and concomitant cell-wall synthesis.

## Discussion

**Figure 5:**
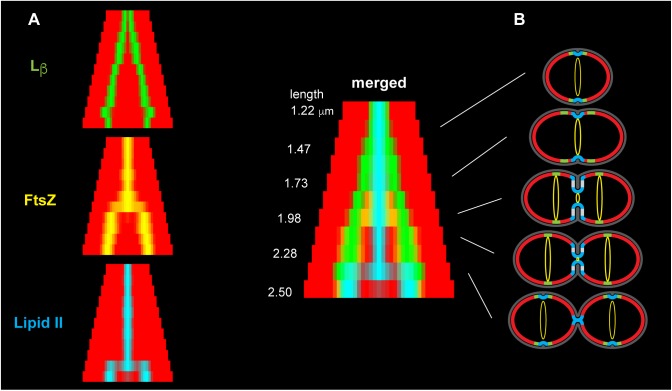
Organization of lipid domains during the cell cycle of *S. pneumoniae*. **(A)** To compare different labeling experiments, cells were grouped in size classes and the fluorescence signals were averaged for each class and symmetrized. The panels show the regions of maximum signal for L_β_-phase (NBD-PG) (green), fluorescent FtsZ (yellow) and lipid II-NBD (cyan), on a background representing the L_α_-phase (red). **(B)** Model of the membrane during the cell cycle of *S. pneumoniae* with the corresponding experimental fluorescence patterns. The cell-wall is in dark gray, the Z-ring is in yellow. L_β_-phase lipids are localized at the equator where the curvature is minimal (green). L_α_-phase bilayer forms the hemispheres where the curvature is larger (red). The cell-wall precursor lipid II is localized at the sites of maximal curvature (cyan) where cell-wall synthesis takes place.

These observations suggest for the cell cycle of ovoid bacteria a simple “chicken-and-egg” model that includes properties of the membrane (Figure 5). To minimize bending stress, L_β_-phase lipids could spontaneously constitute a belt around the equator, while L_α_ membrane forms the hemispheres. The L_β_-phase belt would prevent diffusion of lipids and membrane proteins across the equator, thus enforcing a clear distinction between the parental and novel hemispheres. The lipid composition at the equator, resulting from the cellular geometry, may underlie the recruitment of Z-ring membrane anchors and the division machinery. Constriction of the Z-ring, by inducing large local curvature of the membrane, can then recruit the cell wall precursor lipid II as its dynamic undecaprenyl chain is best suited to accommodate a sharp membrane bending. The regions with the sharpest curvature at the leading edge and the periphery of the septum are indeed thought to be the site of cell-wall synthesis (Tsui et al., 2014).

As the L_β_-phase is characterized by large interaction between individual molecules, splitting of equatorial belt to allow lipid II insertion may require assistance by division proteins. In this respect, we have observed by atomic force microscopy that the amphipathic helix of the division protein FtsA that anchor FtsZ to the membrane (Pichoff and Lutkenhaus, 2005) disrupts condensed domains in mixed phospholipid monolayer (Supplementary Figure S7). This observation suggests that molecules that modify the lamellar phase properties could be developed as antibacterials.

The model proposed here that links the cell division process to cellular geometry through lipid lamellar phase segregation implies the existence of gel phase membrane *in vivo*, which is difficult to demonstrate. Laurdan or Di-4-ANEPPDHQ are fluorescent probes that are sensitive to lipid packing, and spectral microscopy could reveal membrane organization details, provided that sufficient resolution can be obtained to examine the small pneumococcal cells (Sezgin et al., 2015). Direct measurement of the lateral diffusion by Fluorescence Recovery After Photobleaching or Fluorescence Correlation Spectroscopy in the different location of the cell surface (Stricker et al., 2002) could also establish the existence of a gel phase, provided that an adequate probe can be inserted in the membrane at the cellular equator of the pneumococcus. Finally, the level of acyl chain unsaturation in *S. pneumoniae* may be manipulated by tuning the expression of the FabM desaturase (Marrakchi et al., 2002) to explore further the interplay between the membrane and the cell cycle.

## Data Availability

Source images are available at https://figshare.com/s/8ffa51e0a30be7c26ff8.

## Author Contributions

PC was involved in the conceptualization, performed the experiments, and contributed to writing. JJ contributed to the lipid analysis. VV performed by AFM. CD carried out some microscopy observations. AZ acquired funding, performed the red edge excitation shift experiments, interpreted results, and wrote the manuscript.

## Conflict of Interest Statement

The authors declare that the research was conducted in the absence of any commercial or financial relationships that could be construed as a potential conflict of interest.
