# Supplementary material for: Lipid Phases and Cell Geometry During the Cell Cycle of Streptococcus pneumoniae
Source: Front Microbiol. 2019 Mar 18;10:351. doi: 10.3389/fmicb.2019.00351 (PMC6432855; doi:10.3389/fmicb.2019.00351)
Supplement: Supplementary file 1 [file Data_Sheet_1.PDF]

Supplementary Material Figure Legends

Lipid phases and cell geometry during the cell cycle of *Streptococcus pneumoniae*

Philippe Calvez, Juliette Jouhet, Véronique Vié, Claire Durmort & André Zapun*

*Corresponding author:
andre.zapun@ibs.fr

Supplementary FIGURES S1 to S7

Supplementary TABLES S1 to S4

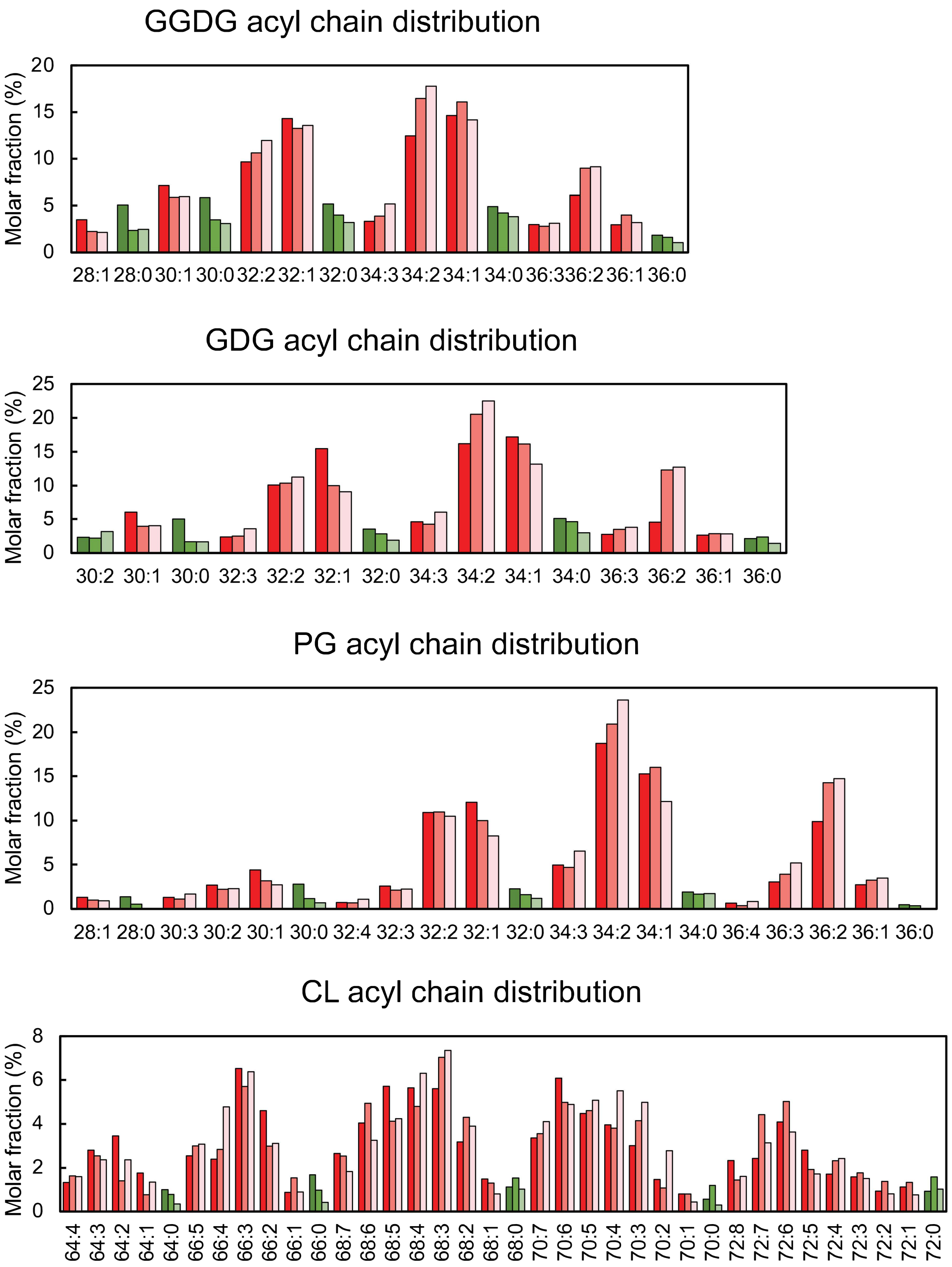

Supplementary FIGURE S1. *S. pneumoniae* membrane composition. Relative amount of fully saturated lipids (green) or lipids with at least one unsaturation (red) with the different head groups. GDG, glucosyldiacylglycerol; GGDG, galactosylglucosyldiacylglycerol; PG, phosphatidylglycerol; and CL, cardiolipin. The x:y nomenclature refers to the total number of carbon atoms per lipid (x) and the total number of unsaturation (y). Lipid analysis was performed during the exponential growth phase (deep coloring), early and late stationary phase (middle and light coloring, respectively). Mass spectrometry data are given in Supplementary Tables S1 to S4.

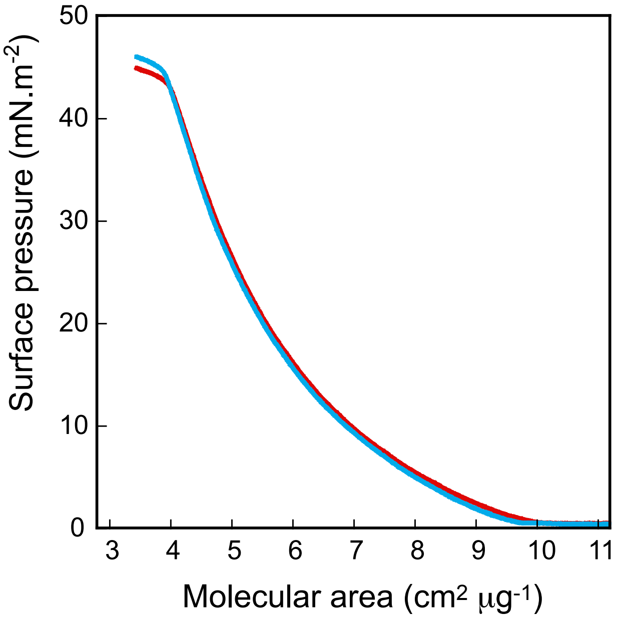

Supplementary FIGURE S2. Surface pressure isotherms of monolayers prepared with lipid extract from cells in late stationary phase recorded at 25°C (cyan) and 43°C (magenta).

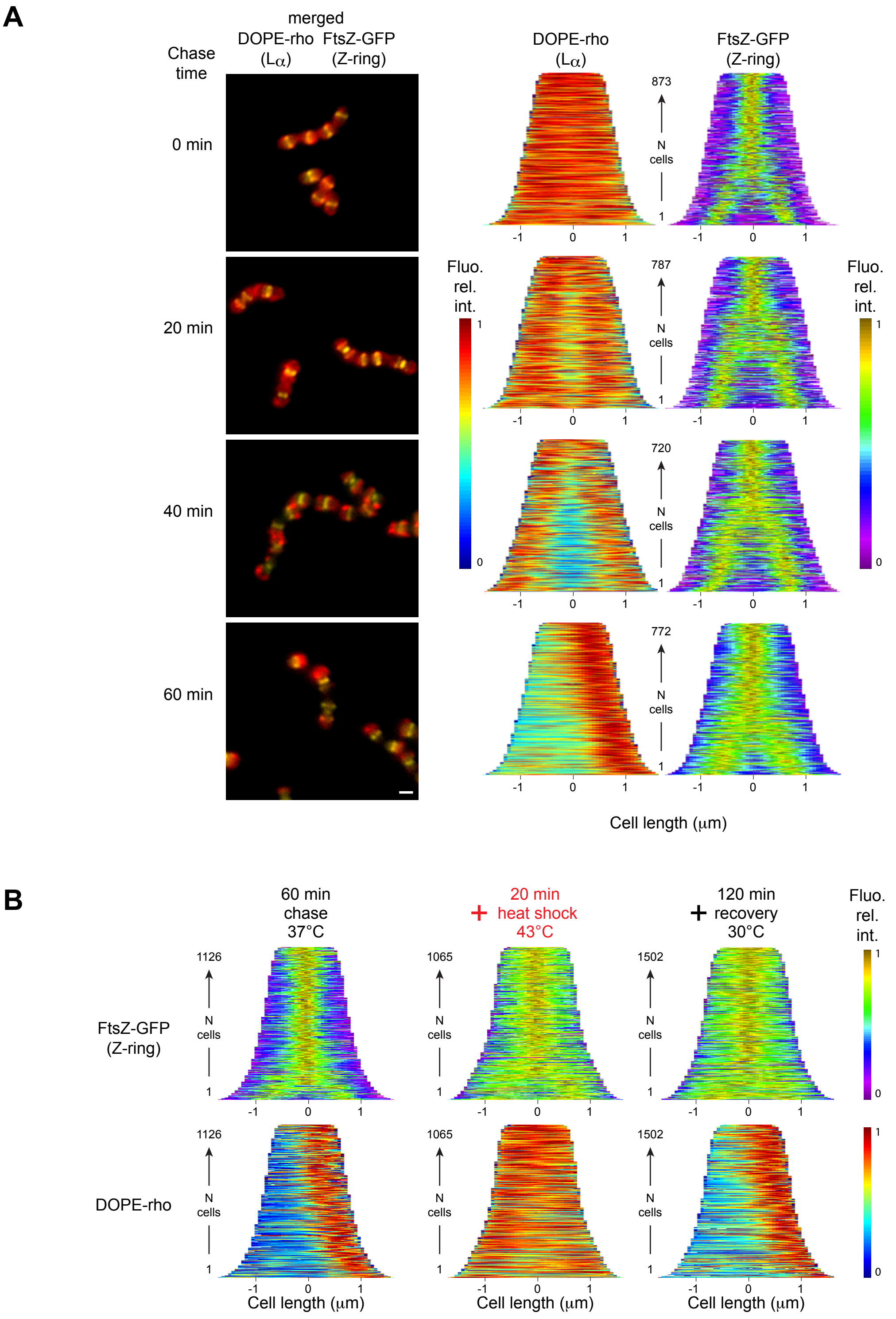

Supplementary FIGURE S3. Localization of potential lipid phases in *S. pneumoniae*. **(A)** Growing cells expressing FtsZ-GFP (yellow) labeled with fluorescent DOPE-rho to reveal L_α-phase (red) imaged after various chase time and the corresponding demographs showing the distribution of the fluorescent signals along the main cell axis in the population. **(B)** Demographs of DOPE-rho-labeled pulse-chased cells (red) expressing FtsZ-GFP (yellow) submitted to a heat-shock prior to recovery. Scale bar is 1 µm.

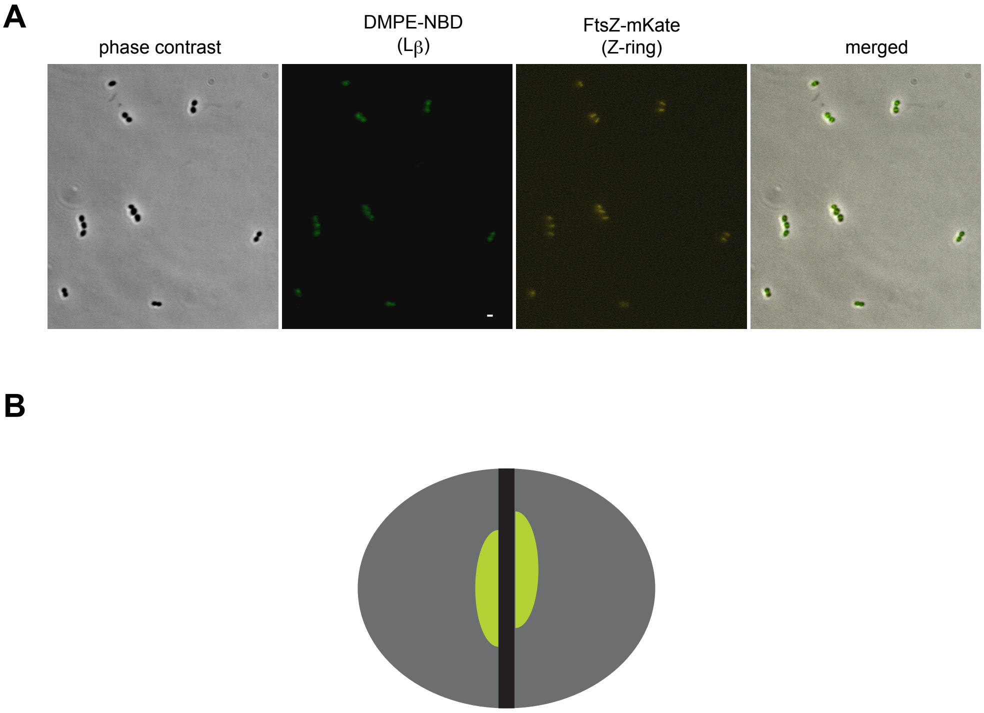

Supplementary FIGURE S4. Localization of potential lipid phases in *S. pneumoniae*. **(A)** Growing cells expressing FtsZ-mKate (red) labeled with fluorescent DMPE-NBD to reveal L_β-phase (green). **(B)** Schematic interpretation of the localization of the NBD-PG signal as domains stacked against an equatorial belt.

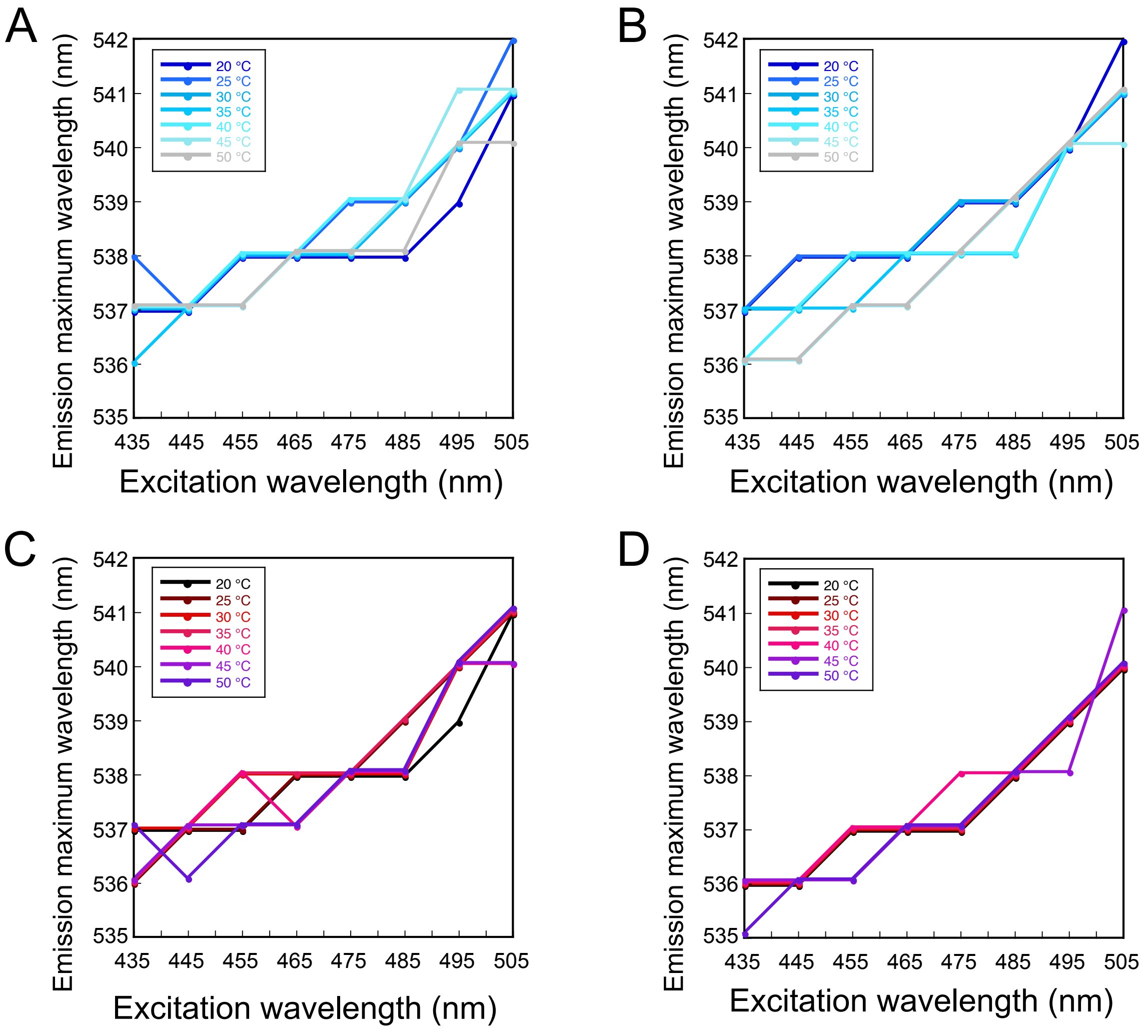

Supplementary FIGURE S5. Red edge excitation shift of NBD-PG at temperatures ranging from 20°C to 50°C, in DPPG **(A, B)** or POPG **(C, D)** vesicles in the absence **(A, C)** or presence of 10 mM $MgCl_2$ **(B, D)**.

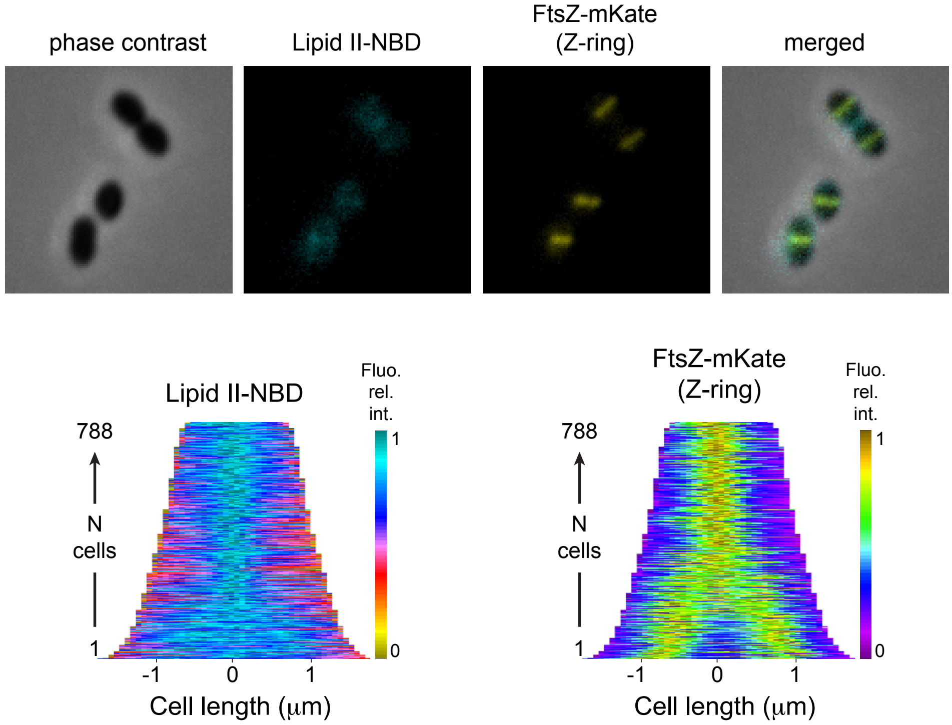

Supplementary FIGURE S6. Lipid II localization in fixed cells. Growing cells expressing FtsZ-mKate (yellow) were fixed with paraformaldehyde prior to labeling with fluorescent lipid II-NBD (cyan) and the corresponding demographs. The scale bar is 1 μm.

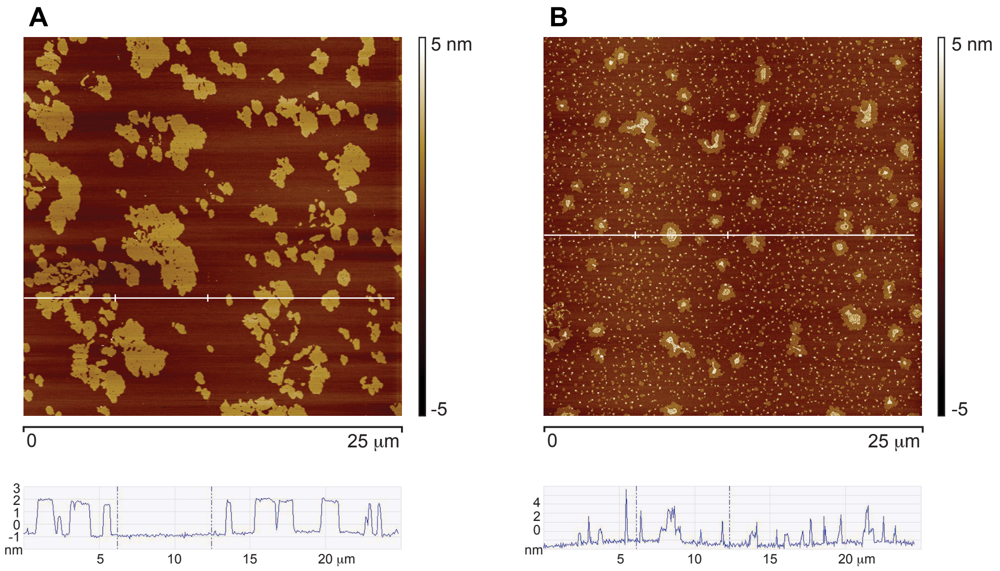

Supplementary FIGURE S7. Effect of FtsA C-terminal helix peptide on monolayer lipid phases. AFM height images of mixed DPPG and DOPG (1:1, w/w) monolayer without **(A)** and with **(B)** added FtsA C-terminal helix. DPPG condensed phase domains appear higher than the DOPG fluid phase domains (background).

Supplementary TABLE S1. Mass and relative amount of the different GDG lipids.

		Exponential		Early stat.		Late stat.	
m/z	Species	Intensity	Molar fract. (%)	Intensity	Molar fract. (%)	Intensity	Molar fract. (%)
852	28:1	36999766	3.5	29517490	2.2	35901869	2.1
854	28:0	53433904	5.1	31203241	2.4	41322420	2.5
880	30:1	75473509	7.1	77510634	5.9	100001865	6.0
882	30:0	61914045	5.9	45760606	3.5	51836583	3.1
906	32:2	102182191	9.7	139604681	10.6	200037617	12.0
908	32:1	151192968	14.3	174069584	13.3	226933927	13.6
910	32:0	54736702	5.2	52370256	4.0	54037361	3.2
932	34:3	35231183	3.3	51097255	3.9	86873704	5.2
934	34:2	131626661	12.5	216142619	16.5	297138752	17.8
936	34:1	154646648	14.6	211272389	16.1	236857005	14.2
938	34:0	52025373	4.9	55740338	4.2	64401895	3.9
960	36:3	31564112	3.0	36830181	2.8	52267662	3.1
962	36:2	64580921	6.1	118131671	9.0	152977623	9.1
964	36:1	31351719	3.0	52573420	4.0	53508800	3.2
966	36:0	19743639	1.9	21309381	1.6	17887085	1.1
	Total GGDG	1056703341	100	1313133746	100	1671984168	100

Supplementary TABLE S2. Mass and relative amount of the different GGDG lipids.

		Exponential		Early stat.		Late stat.	
m/z	Species	Intensity	Molar fract. (%)	Intensity	Molar fract. (%)	Intensity	Molar fract. (%)
716	30:2	35623056	2.3	36708124	2.2	90397728	3.2
718	30:1	92599144	6.0	65976872	4.0	115137880	4.0
720	30:0	77029232	5.0	27820070	1.7	47149528	1.7
742	32:3	36398428	2.4	41713936	2.5	102229848	3.6
744	32:2	154124704	10.1	172440704	10.3	320155424	11.2
746	32:1	236549776	15.4	166314528	10.0	258141680	9.1
748	32:0	54375928	3.5	47428060	2.8	53047432	1.9
770	34:3	70773200	4.6	71087032	4.3	172186176	6.0
772	34:2	247768688	16.2	342463168	20.5	640905984	22.5
774	34:1	263116208	17.2	269087424	16.1	374701920	13.1
776	34:0	78375984	5.1	77386232	4.6	85169984	3.0
798	36:3	42291096	2.8	58344924	3.5	108381920	3.8
800	36:2	69883288	4.6	204935984	12.3	361961856	12.7
802	36:1	40486196	2.6	47688884	2.9	80627280	2.8
804	36:0	32992272	2.2	39318208	2.4	39899500	1.4
	Total GDG	1532387200	100	1668714150	100	2850094140	100

Supplementary TABLE S3. Mass and relative amount of the different PG lipids.

		Exponential		Early stat.		Late stat.	
m/z	Species	Intensity	Molar fract. (%)	Intensity	Molar fract. (%)	Intensity	Molar fract. (%)
663	28:1	8656383	1.3	6262887	1.0	10699131	0.9
665	28:0	9124534	1.4	3526215	0.6	678207	0.1
687	30:3	8627931	1.3	6995346	1.1	19584534	1.7
689	30:2	17814598	2.7	13921732	2.2	26671614	2.3
691	30:1	29154574	4.4	19935068	3.2	31697028	2.7
693	30:0	18524670	2.8	7333711	1.2	8053431	0.7
713	32:4	4797255	0.7	4280515	0.7	12723943	1.1
715	32:3	17088548	2.6	13313087	2.1	26092910	2.2
717	32:2	72123664	10.9	68760304	11.0	121990016	10.5
719	32:1	79755640	12.1	62650268	10.0	96060656	8.2
721	32:0	14860970	2.2	9973542	1.6	14175947	1.2
743	34:3	32812278	5.0	29460828	4.7	76113512	6.5
745	34:2	123813184	18.7	131105728	20.9	274957536	23.6
747	34:1	101009560	15.3	100436520	16.0	141375360	12.1
749	34:0	12533936	1.9	10563037	1.7	20242472	1.7
769	36:4	4321141	0.7	2304376	0.4	9774739	0.8
771	36:3	20166856	3.0	24609932	3.9	60506944	5.2
773	36:2	65333428	9.9	89507896	14.3	171467344	14.7
775	36:1	18068664	2.7	20398320	3.3	40582512	3.5
777	36:0	3075988	0.5	2165354	0.3	960512	0.1
	Total PG	661663802	100	627504666	100	1164408348	100

Supplementary TABLE S4. Mass and relative amount of the different CL lipids.

		Exponential		Early stat.		Late stat.	
m/z	Species	Intensity	Molar fract. (%)	Intensity	Molar fract. (%)	Intensity	Molar fract. (%)
1344	64:4	244926	1.3	314320	1.6	628360	1.6
1346	64:3	517001	2.8	491197	2.5	931715	2.4
1348	64:2	637105	3.5	271002	1.4	931273	2.4
1350	64:1	324493	1.8	148172	0.8	530100	1.3
1352	64:0	186444	1.0	151750	0.8	134305	0.3
1370	66:5	469485	2.5	578591	3.0	1213024	3.1
1372	66:4	441174	2.4	547881	2.8	1884546	4.8
1374	66:3	1205148	6.5	1102958	5.7	2516588	6.4
1376	66:2	850052	4.6	576927	3.0	1226851	3.1
1378	66:1	161535	0.9	297170	1.5	351954	0.9
1380	66:0	310475	1.7	186330	1.0	164641	0.4
1394	68:7	489238	2.6	489397	2.5	720162	1.8
1396	68:6	746041	4.0	954606	4.9	1282505	3.3
1398	68:5	1055364	5.7	796513	4.1	1671615	4.2
1400	68:4	1042219	5.6	927096	4.8	2489240	6.3
1402	68:3	1035084	5.6	1359999	7.0	2900937	7.4
1404	68:2	586224	3.2	831067	4.3	1537221	3.9
1406	68:1	274143	1.5	250991	1.3	316911	0.8
1408	68:0	205340	1.1	295190	1.5	401253	1.0
1422	70:7	620080	3.4	686801	3.6	1619269	4.1
1424	70:6	1123556	6.1	962761	5.0	1928390	4.9
1426	70:5	826183	4.5	890270	4.6	2003751	5.1
1428	70:4	730468	4.0	735357	3.8	2173749	5.5
1430	70:3	554924	3.0	801186	4.1	1966242	5.0
1432	70:2	270308	1.5	207929	1.1	1094791	2.8
1434	70:1	148185	0.8	154678	0.8	171859	0.4
1436	70:0	103303	0.6	229812	1.2	113752	0.3
1448	72:8	429539	2.3	278185	1.4	633412	1.6
1450	72:7	447472	2.4	854474	4.4	1236693	3.1
1452	72:6	754596	4.1	970664	5.0	1431825	3.6
1454	72:5	517059	2.8	370892	1.9	676774	1.7
1456	72:4	315234	1.7	448178	2.3	954630	2.4
1458	72:3	292021	1.6	341285	1.8	594731	1.5
1460	72:2	172142	0.9	265669	1.4	318098	0.8
1462	72:1	207019	1.1	257141	1.3	300279	0.8
1464	72:0	171648	0.9	303190	1.6	408056	1.0
	Total CL	18465228	100.0	19329629	100.0	39459502	100.0
